# ***Aggregatibacter actinomycetemcomitans*** **exacerbates colitis and perturbs the gut microbiota in a murine model​**

**DOI:** 10.1080/20002297.2026.2613536

**Published:** 2026-01-11

**Authors:** Xu Chen, Xiaoming Zhu, Yiran Liu, Lu Ma, Lu Li, Jitong Dong, Zirui Li, Tianyao Wang, Juan Zhang, Yan Xu

**Affiliations:** a State Key Laboratory Cultivation Base of Research, Prevention and Treatment for Oral Diseases, Nanjing Medical University, Nanjing, China; b Department of Periodontology, the Affiliated Stomatological Hospital of Nanjing Medical University, Nanjing, China; c Jiangsu Province Engineering Research Center of Stomatological Translational Medicine, Nanjing, China; d Department of Stomatology, The Fourth Affiliated Hospital of Soochow University; Dushu Lake Hospital; Medical Center of Soochow University, Suzhou, China; e Department of Pediatric and Preventive Dentistry, the Affiliated Stomatological Hospital of Nanjing Medical University, Nanjing, China

**Keywords:** Colitis, gut microbiota, *Aggregatibacter actinomycetemcomitans*, barrier integrity, inflammatory factors

## Abstract

**Background:**

Chronic intestinal inflammation is a hallmark of inflammatory bowel disease (IBD). Studies suggest that salivary bacteria associated with periodontitis may exacerbate colitis, but the specific contributory microbes and mechanisms remain unclear.

**Patients and methods:**

We analyzed stool samples from 28 IBD patients and 21 controls. Bacterial DNA was extracted and quantified using qPCR targeting *Aggregatibacter actinomycetemcomitans* (*A. actinomycetemcomitans*). In a murine model, C57BL/6 mice received DSS followed by daily oral gavage with *A. actinomycetemcomitans*. Disease activity, histopathology and the gut microbiota were evaluated.

**Results:**

Oral administration of *A. actinomycetemcomitans* exacerbated inflammatory symptoms in DSS-induced colitis. The bacterium was detectable in intestinal tissue transiently following high-dose oral administration during the inflammatory phase. Following high-dose gavage, bacterial DNA was transiently detectable in intestinal tissue during inflammation. This treatment was associated with reduced expression of the tight junction protein ZO-1 and mucin MUC-2, elevated inflammatory mediators, and altered gut microbial community structure, including an expansion of taxa associated with dysbiosis.

**Conclusion:**

In a murine colitis model, *A. actinomycetemcomitans* exposure was associated with worsened disease severity, coinciding with impaired barrier integrity, heightened inflammation and gut microbiota alterations. These exploratory findings highlight the potential role for specific periodontal microbes in modulating intestinal inflammation and warrant further investigation.

## Introduction

Oral diseases are a global public health challenge, affecting more than 3.5 billion people worldwide [1]. Periodontal disease (PD) is one of the most common oral disorders, posing a substantial health burden in both developed and developing countries [2,3]. PD comprises a group of oral infectious conditions caused by bacterial biofilms, primarily including periodontitis and gingivitis. At the onset of the disease, an ecological imbalance in the host leads to disturbance of the host–microbiome equilibrium, promoting the proliferation of specific periodontal pathogens in saliva [4,5]. The extent to which oral microbes reach and successfully colonize the distal intestine has been controversial [6–8]. Nevertheless, the daily swallowing of a substantial volume of saliva establishes a physical connection between the oral cavity and the colon, the two ends of the digestive tract [6,9].

In parallel, periodontitis has been associated with various systemic diseases, including inflammatory bowel disease (IBD) [10]. IBD is characterized by recurrent chronic inflammation of intestinal tissue and primarily encompasses ulcerative colitis (UC) and Crohn's disease (CD). Its pathogenesis is complex and multifactorial, involving interactions among environmental, genetic and microbial factors with the host immune system, leading to dysregulated immune responses [11]. The role of intestinal microbiota dysbiosis and its immunomodulatory implications in IBD represents a growing area of extensive research and clinical interest, with specific bacterial species being closely related to IBD pathogenesis [12]. Interestingly, ecological disruptions in the oral microbiome form a network connecting oral infection with intestinal inflammation [13]. Our previous studies have also identified a microbiological association between periodontitis and CD [14]. In fact, potential pathogenic microbes may act as a link between these two diseases [15].

The complex symbiotic relationship between the host and microorganisms is crucial for maintaining human health. Both IBD and periodontitis are characterized by disruption of the host-microbiota network [5,16]. In periodontitis, oral dysbiosis leads to the expansion of pathogenic bacteria, which may subsequently contribute to gut microbial dysbiosis and exacerbate intestinal inflammation in mammals [10,17]. This process may be related to the periodontitis-associated pathogens in saliva [18]. However, while changes in the gut microbiota and related pathogenic mechanisms have been extensively characterized, the extent and precise mechanisms by which periodontal pathogens in the oral cavity, the opposite end of the digestive niche, promote intestinal inflammation remain incompletely understood.

Here, we observed a significant enrichment of *Aggregatibacter actinomycetemcomitans* (abbreviated as *A. actinomycetemcomitans*) in the fecal samples of patients with IBD. This bacterium is a periodontal pathogen associated with localized aggressive periodontitis (LAgP) [19]. However, the direct role of *A. actinomycetemcomitans* in intestinal inflammation remains unclear. Therefore, this study aimed to investigate whether and how exposure to *A. actinomycetemcomitans* influenced the development and severity of colitis.

## Patients and methods

**Figure:**
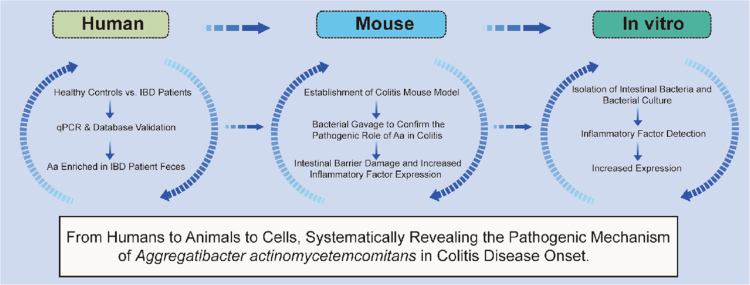


### Patients and stool samples collection

It is important to noted that the human component of this study was designed as an exploratory, hypothesis-generating analysis. Given this exploratory aim, detailed clinical phenotyping – including IBD subtype classification (ulcerative colitis vs. Crohn’s disease), comprehensive periodontal assessment, detailed timelines of medication use (antibiotics, biologics, immunosuppressants, steroids), oral health parameters, and rigorous matching of controls for factors such as age, sex and smoking status – was not systematically performed.

Mouse feces were collected either before or during dissection under controlled conditions and similarly preserved at –80 °C until subsequent processing. Human stool samples were obtained from the Affiliated Hospital of Stomatology, Nanjing Medical University (28 IBD patients and 21 non-IBD controls). The research was conducted in accordance with the World Medical Association Declaration of Helsinki, and all participants provided written informed consent. Patient recruitment was conducted at The Affiliated Stomatological Hospital of Nanjing Medical University between 2016 and 2019. The study protocol was approved by the Clinical Research Ethics Committee of the Affiliated Hospital of Stomatology, Nanjing Medical University (PJ2021-014-01). The essential demographic and clinical characteristics of the participants were summarized in Table S1. Fecal samples were collected from all participants using sterile, DNA-free collection kits. The participants were instructed to immediately freeze the samples at –20 °C after collection. The frozen samples were then transported to our laboratory on dry ice and stored at –80 °C until DNA extraction. All handling and processing steps were documented to maintain the chain of custody.

## *In vitro* experiments and sample analysis

### Extraction of bacterial DNA from feces and *A. actinomycetemcomitans* quantification by quantitative polymerase chain reaction (qPCR)

To extract bacterial DNA from feces, we thawed human or mouse fecal samples that had been stored at −80 °C on ice. We subsequently used the QIAamp PowerFecal Pro DNA Kit (QIAGEN, Germany) to extract microbial genomic DNA from all the samples. The concentration and purity of the extracted DNA were measured using a NanoDrop 2000 spectrophotometer (Thermo Fisher Scientific, USA), and the DNA was diluted and stored according to its concentration. Quantitative PCR was performed using SYBR Premix Ex Taq II (Takara, Japan). The relative abundance of *A. actinomycetemcomitans* was assessed via the *ropB* gene, where 1  μL of bacterial DNA at a concentration of 1  μg/μL was added to a 10  μL qPCR reaction system, and the ΔCt method was applied for calculation [20]. The Ct values of *A. actinomycetemcomitans* were normalized using universal primers targeting total bacteria [21]. The primers were listed in Table S2.

### DNA gel electrophoresis

To assess the recovery of orally administered bacteria from the intestinal tract, the DNA of all bacteria cultivated on streptomycin-resistant plates was extracted according to the manufacturer's instructions (TIANamp Bacteria DNA Kit, China), and then amplified segments of the *A. actinomycetemcomitans ropB* and *ltxA* genes. The PCR results were loaded onto a 1% agarose gel (Biowest, Spain) and electrophoresed in 1x TAE electrophoresis buffer (Sangon Biotech, China). Bands were revealed. The primer sequences are shown in Table S2.

### Determination of total bacterial biomass

The total bacterial biomass was determined based on optical density using LIVE/DEAD BacLight Bacterial Viability kits (L13152, Invitrogen, USA) consistent with the manufacturer's instructions. Bacterial cultures in the logarithmic growth phase were added to BHI broth at a ratio of 1:50 with or without the addition of enteric bacterial extracts, and the fluorescence intensity of live bacteria (green, OD_530_, F_cell,em1_) and dead bacteria (red, OD_630_, F_cell,em2_) were measured, respectively after overnight incubation. The data were analyzed by the ratio_G/R_ = F_cell,em1_/F_cell,em2_.

### Cell culture

NCM460 and NB4 cells were obtained from the cell lines preserved in the Key Laboratory of Oral Diseases of Jiangsu Province, cultured in high-glucose DMEM and 1640 basic medium, respectively (GIBCO, USA), supplemented with 10% fetal bovine serum (FBS, BI, Israel) and 1% streptomycin–penicillin (NCM, China), and incubated at 37 °C in a humidified atmosphere with 5% CO_2_. Prior to co-cultured with bacterial extracts, the cells were switched to a culture medium devoid of penicillin/streptomycin. The cells were seeded in 6-well plates at a density of 2 × 10^5^ cells per well in 2 mL of complete medium, or in 96-well plates at a density of 1 × 10^4^ cells per well in 100  μL of complete medium, and allowed to adhere overnight prior to treatments. The readouts mainly included the expression of inflammatory cytokines (measured by ELISA).

## *In vivo* animal experiments

### Preparation of bacterial strains for gavage

Bacterial cultures used for oral gavage in mouse experiments were prepared as follows. *A. actinomycetemcomitans* (ATCC 29523) strain was grown on Colombian blood agar plates (Comagal, Shanghai) for 2 days at 37 °C in a 5% CO_2_ incubator; *P. gingivalis* W83 was cultured for 3 days at 37 °C on brain heart infusion (BHI; OXOID) agar plates supplemented with vitamin K (10 mg·mL^−1^), chlorohemin (5 mg·mL^−1^ hemin, 10 μmol NaOH) and 10% defibrinated sheep blood in an anaerobic incubator. For routine culture, avirulent *E. coli* MG1655 and *A. actinomycetemcomitans* were cultured in a constant temperature shaker at 37 °C under normoxic conditions. *E. coli* was cultured in LB broth containing tryptone (10 mg·mL^−1^), yeast (5 mg·mL^−1^) and NaCl_2_ (10 mg·mL^−1^) for about 6 h while *A. actinomycetemcomitans* was cultured in BHI broth overnight. For experiments requiring antibiotic selection, streptomycin-resistant *A. actinomycetemcomitans* was generated by acclimation in BHI broth containing increasing concentrations of streptomycin (from 0.1 μg·mL^−1^ to 50 μg·mL^−1^) over approximately 30 days, with concentration increments every other day.

### Preparation of gut bacterial extracts

The intestinal bacterial extract was prepared as described previously [22]. Briefly, 0.5 mg of feces from *A. actinomycetemcomitans*-gavaged colitis mice was collected and suspended in 1.5  ml of sterile PBS, shaken and homogenized for pelleting, then passed through a 40 μm filter (Falcon, USA) three times in a sterile Petri dish and washed twice by centrifugation in sterile PBS (8300 × g, 5 min). The pellet was then resuspended in 200 μl of sterile PBS containing protein hydrolase inhibitor (Keygen Biotech, China) and phosphatase inhibitor (Keygen Biotech, China). Bacterial inactivation was performed by incubation in a water bath (65 °C, 1 h). The suspension was sonicated on ice (four cycles of 30 s on, 30 s off) [23], and the protein concentration was assessed. The sterility of the extracts was confirmed prior to use. For co-cultured with cells, bacterial extracts were added to the culture system at a final concentration of 1 μg/ml. The treatment did not significantly affect cell viability (Figure S5).

### Animal experiments

We used random allocation with blinding of the outcome assessors. SPF C57BL/6 wild-type (WT) mice were purchased from the Model Animal Center of Nanjing Medical University. Upon arrival, the mice were randomly assigned to cages, and the cages were then randomly allocated to different treatment groups to minimize cage effects, especially given the microbiome-focused outcomes. All the mice were housed, and experiments were conducted at the specific pathogen-free (SPF) animal facility of the Animal Core Facility of Nanjing Medical University under controlled conditions (temperature: 22 ± 1°C, humidity: 50 ± 10%, 12-h light/dark cycle). All animal experiments were conducted in accordance with the guidelines approved by the Institutional Animal Care and Use Committee (IACUC) of Nanjing Medical University (Approval Number: IACUC-2102014). The DSS-induced colitis model and inflammation assessment were performed as described [24,25]. Six- to eight-week-old mice were treated for 5‒6 days with 2.5% or 3% DSS (MP Biomedicals, USA), followed by 2‒3 days of normal drinking water. A single batch of dextran sulfate sodium (DSS; molecular weight 36–50 kDa, MP Biomedicals, catalog #160110) was used for all experiments to ensure uniformity. A stock solution was freshly prepared for each experiment by dissolving DSS in autoclaved drinking water to the specified concentration (2.5% or 3% w/v).

For oral pathogenic bacteria colonization and toxicity test, the mice were administered *A. actinomycetemcomitans* or *P. gingivalis* every day, a negative control bacteria or PBS vector was used, and the dose was 1 × 10^9^ CFU every time [17]. Weight loss, fecal consistency, and intestinal bleeding were evaluated on a daily basis during the trial, and these data were used to calculate the disease activity index (as given in Table S3), with a total score ranging from 0 to 12. The group for *P. gingivalis* was established concurrently with each experimental group, although experiments involving *P. gingivalis* and *A. actinomycetemcomitans* were conducted in separate and temporally distinct trials to avoid any potential cross-contamination or interaction.

To create a microbiota-depleted model, the mice received a broad-spectrum antibiotic cocktail (AMP 0.2 g/L, VAN 0.1 g/L, NEO 0.2 g/L and MTZ 0.2 g/L) ad libitum in the drinking water for 3 consecutive days. Antibiotic water was then replaced with normal drinking water for a 24-h washout period prior to the initiation of DSS treatment and bacterial gavage.

To ensure consistent bacterial inoculation at each gavage, the bacterial suspension was verified by the plate counting method for CFU prior to administration. The concentration of the bacterial suspension used for each gavage was adjusted to the required low concentration, with the actual inoculated dose controlled within a 10% margin of error.

### Collection of mouse samples

Mouse feces were collected either before or during dissection under controlled conditions and stored at –80 °C until processing. For RNA and histological analysis, colonic tissues were collected upon sacrifice.

### Isolation and identification of fecal bacteria

Isolate fecal bacteria as previously described [21,26]. To isolate and identify bacteria from mouse feces, two fresh fecal particles were immediately homogenized in 1 ml of BHI broth containing 5% FBS. After gradient dilution, the homogenate was inoculated onto BHI agar plates containing 50 μg/ml streptomycin. The use of streptomycin-containing plates was used to selectively assess the recovery of the streptomycin-resistant, orally administered *A. actinomycetemcomitans* from the intestinal tract. The plates were incubated in a 5% CO_2_ incubator at 37 °C for 48–72 h. Single colonies were picked, re-streaked for purity, and subjected to genomic DNA isolation. Bacterial identification was performed by amplifying the 16S rRNA gene using primers listed in Table S2, followed by Sanger sequencing (Sangon Biotech, Shanghai) and NCBI BLAST (Basic Local Alignment Search Tool) analysis (Figure S1, Table S1).

### RNA extraction and inflammatory cytokine expression analysis in colon tissue

To analyze gene expression in mouse colonic tissue, RNA was extracted from dissected tissues immediately chilled in liquid nitrogen. The tissue was homogenized in 1 ml of TRIzol (Thermo Fisher, USA) using a mortar and pestle, and total RNA was extracted using the RNA Simple Total Kit (TianGen, China). To mitigate potential interference from DSS in subsequent PCR, 0.01 g/L spermine (Aladdin, China) was added during the reverse transcription reaction using PrimeScript™ RT Master Mix (TaKaRa, Japan), as this concentration does not affect the reaction [27]. Quantitative PCR was performed using SYBR Premix Ex Taq II (TaKaRa, Japan) and gene-specific primers (Table S2). The comparative CT method (2^−ΔΔCt^) was used to calculate relative gene expression levels, with Gapdh serving as the internal control.

### Pathology assessment

Mice were euthanized by cervical dislocation under isoflurane anesthesia (induction at 4% and maintenance at 1.5–2% in 100% oxygen). Colon specimens were then collected, formalin-fixed and paraffin-embedded for histological examination. The 4 μm sections were stained with hematoxylin and eosin (H&E), and the histological scores were scored by two experimenters who were blinded to the group information [28]. The intraclass correlation coefficient (ICC) was used to evaluate inter-rater reliability, with an overall ICC greater than 0.75, indicating excellent agreement for all histological parameters. Specifically, the degree of inflammation in each segment of intestinal tissue was evaluated independently (0, none; 1. low density is restricted to the mucous membrane. 2, moderate to high mucosal density and/or submucosa with low to moderate density; 3, submucosal extension into the myometrium; 4, high density with frequent wall penetration); and epithelial/crypt damage (0, none; 1, 1/3 of the base; 2, 2/3 of the base; 3, absence of crypt; 4, crypt and surface epithelial destruction). Each variable was multiplied by a factor reflecting the percentage of colon involved (1, 0–25%; 2, 26–50%; 3, 51–75%; and 4, 76–100%). The scores assigned to each variable were added together to produce an overall score.

### Immunofluorescence staining

The ZO-1 and MUC-2 protein-targeting rabbit antibodies (Servicebio, China) were applied to the 4 μm-thick tissue sections, which were subsequently incubated overnight at 4 °C, washed with phosphate-buffered saline, and then treated with anti-rabbit fluorescent secondary antibodies (Servicebio, China) at 37 °C for 1 h. Following washing, the tissue nuclei were re-stained with 4-methyl-6-methyl-2-phenylindole (DAPI). Utilized a fluorescence microscope to scan the area, and take pictures while the eye was kept out for the tissue's fluorescence.

### Fluorescence in situ hybridization (FISH)

Tissue sections were probed with an appropriate concentration of *A. actinomycetemcomitans* 16S rRNA-specific oligonucleotide 5’-TCCATAAGACAGATTC-3’ labeled with Cy3 dye (Servicebio, China). Each slide was rinsed with aseptic washing solution and restained with DAPI after being incubated overnight in a dark moist environment. A fluorescent microscope was used to examine the images.

### Enzyme-linked immunosorbent assay (ELISA)

The eyeballs were enucleated when the mice were sacrificed, the peripheral blood of each group was collected, and then centrifuged at a speed of 5000 × g for 15 min to separate the serum. The concentrations of cytokines in serum of different groups of mice, including II-1β, IL-6 and TNF-*α*, were measured using the enzyme-linked immunosorbent assay (ELISA) kit (Neobioscience, China) in accordance with the manufacturer's instructions. The same supernatant inflammatory factor assay was performed on cultured cells. The samples were analyzed in triplicate, and the concentrations were calculated according to the standard curve.

### Statistical analysis

All the statistical analyses were performed using SPSS, GraphPad Prism, and R. For the longitudinal measurements, the raw values were first normalized by calculating the relative change from each subject’s baseline (Day 0) to ensure independence across the time points. These normalized values were then treated as independent observations for cross‑sectional comparisons at individual time points.

For comparisons among multiple groups, one‑way ANOVA was applied. When ANOVA indicated significant differences, post‑hoc pairwise comparisons were conducted using Fisher’s least significant difference (LSD) test. In cases where the assumption of homogeneity of variances was violated (as assessed by Levene’s test), Dunnett’s T3 test—which does not require equal variances—was used instead. Comparisons between two independent groups were performed using the unpaired Student’s t‑test.

For microbiome alpha‑diversity indices, the Kruskal–Wallis rank‑sum test followed by Dunn’s post–hoc test was applied. Beta‑diversity differences between groups were assessed using the ANOSIM (analysis of similarities) method.

Data are presented as mean ± SEM unless otherwise noted. A two‑tailed *P* < 0.05 was considered statistically significant.

## Results

### *A. actinomycetemcomitans* was significantly enriched in fecal samples from IBD patients

**Figure 1.**
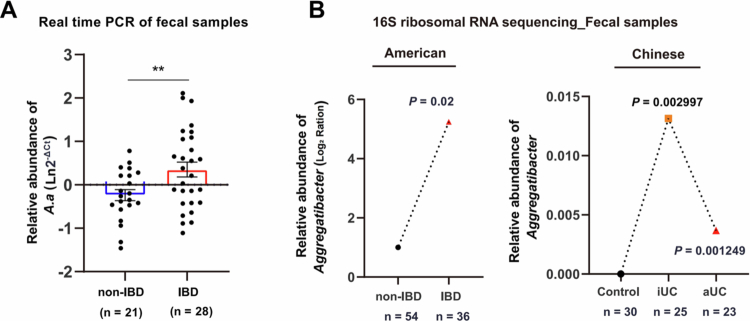
*A. actinomycetemcomitans* was enriched in the stool of IBD patients. (A) Real-time qPCR analysis of fecal *A. actinomycetemcomitans* levels in Chinese IBD patients (*n* = 28) and non-IBD participants (*n* = 21). (B) *A. actinomycetemcomitans* levels in stool samples from two public cohorts of Americans (IBD: *n* = 36; non-IBD: *n* = 54) and Chinese (iUC: *n* = 25; aUC: *n* = 23; healthy control: *n* = 30) by 16S ribosomal RNA sequencing*.* Data are representative of two independent experiments. The two groups are compared by two-sided unpaired Student’s t-test (Levin test for homogeneity of variance),*p < 0.05, **p < 0.01, ***p < 0.001. Differences in means ( ± SEM) are displayed.

As an exploratory analysis to identify potential microbial links, we initially screened for the differences in the abundance of *A. actinomycetemcomitans* in a recruited fecal metagenomic cohort, including 28 IBD patients and 21 people without IBD (non-IBD). The results showed a significant enrichment of *A. actinomycetemcomitans* in fecal samples from IBD patients (*P* < 0.01) (Figure 1A).

*A. actinomycetemcomitans* is an *Aggregatibacter* species [29], through the analysis of fecal microbial sequencing data from two IBD study cohorts from the United States [30] and China [31] (US: 54 non-IBD patients and 36 IBD patients, *P* = 0.02; Chinese: 48 UC patients and 30 healthy subjects, *P* < 0.01), we determined the enrichment of *Aggregatibacter* in IBD patients compared with the control group (Figure 1B).

### Oral administration of *A. actinomycetemcomitans* exacerbated the severity of DSS-induced colitis

**Figure 2.**
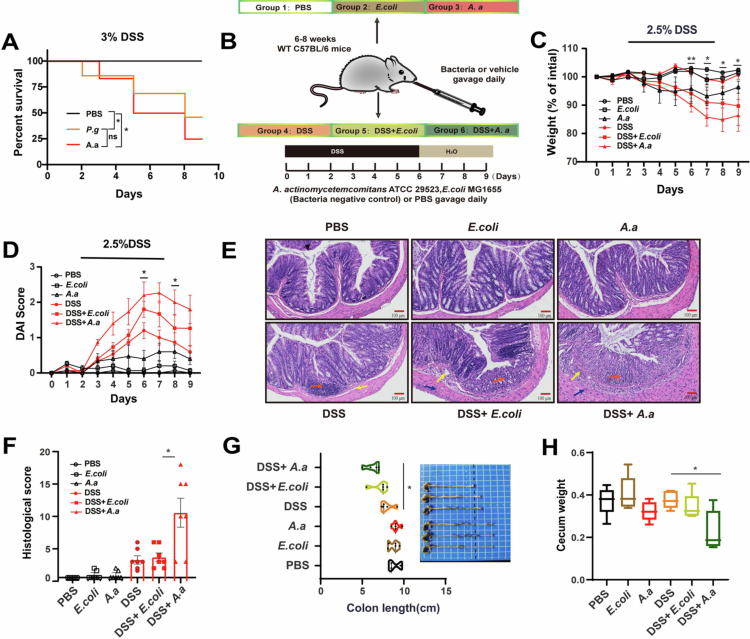
Exposure to *A. actinomycetemcomitans* was associated with exacerbated colitis symptoms in SPF mice. (A) Kaplan‒Meier survival curves of mice co-administered 3% DSS with *A. actinomycetemcomitans* ATCC 29523 or *P. gingivalis* W83. (B) Schematic of the experimental design and timeline. (C) Body weight and (D) disease activity index (DAI) of each group. (E) Representative colonic histological images (scale bar, 100 μm). Blue arrows: inflammatory cell infiltration; yellow arrows: epithelial damage; orange arrows: crypt distortion. (F) Histological score indicating colitis severity. Scores were derived from the assessment of at least 3 longitudinally-oriented crypt-villus fields per mouse. (G) Colon length and (H) cecum weight measured at day 9 post-inoculation. Data are presented as mean ± SEM and are representative of two independent experiments. Each symbol in (C), (D), (F), (G), and (H) represents one mouse (*n* = 5 mice per group). The graphic typically depicts the level of variability compared with DSS + *A.a* group with DSS group, *p < 0.05, **p < 0.01, ***p < 0.001, by one-way ANOVA followed by the LSD post hoc test or two-sided unpaired Student’s t-test and Dunnett T3 (Uneven variance). The Shapiro‒Wilk test for data normality and the Levin test for homogeneity of variance were used. ANOVA, analysis of variance; DSS, dextran sodium sulphate; LSD, least significant difference.

People normally swallow about 1.5 L of saliva every day [32], and we hypothesized that the increased abundance of *A. actinomycetemcomitans* in the feces of IBD patients originated from the daily swallowing of saliva. Previous studies demonstrated the promoting effect of *Porphyromonas gingivalis* (*P. gingivalis*) on colitis [33,34]. Therefore, in a colitis model caused by 3% DSS, we simultaneously overgavaged *P. gingivalis* and *A. actinomycetemcomitans*. According to survival analysis curves, *A. actinomycetemcomitan* exhibited a greater mortality rate than *P. gingivalis* (Figure 2A), which directly validated the toxicity of *A. actinomycetemcomitan* and may increase the development of colitis.

There are competitive microbes in the intestines of normal IBD patients. To investigate whether oral ingestion of *A. actinomycetemcomitans* could exacerbate intestinal inflammation, we induced colitis in SPF mice with 2.5% DSS and simulated gastrointestinal exposure by routine daily intragastric administration of *A. actinomycetemcomitans*, the non-pathogenic control *Escherichia coli* MG1655 [35] or a PBS vehicle (Figure 2B). Comparison of weight loss and disease activity index (DAI) scores revealed that *A. actinomycetemcomitans* administration significantly exacerbated colitis in SPF mice compared to control groups (Figure 2C and D). Measurements of intestinal length, cecal weight, H&E staining, and histological scores further demonstrated that, compared to the PBS gavage group and the non-pathogenic control *E. coli* MG1655 group, *A. actinomycetemcomitans* exerted a more pronounced pro-colitic effect in DSS-induced colitis (Figure 2E–H). Collectively, our results indicated that the oral administration of *A. actinomycetemcomitans* exacerbated the severity of DSS-induced colitis in mice.

### *A. actinomycetemcomitans* DNA was transiently detectable in the colon following oral gavage during colitis

**Figure 3.**
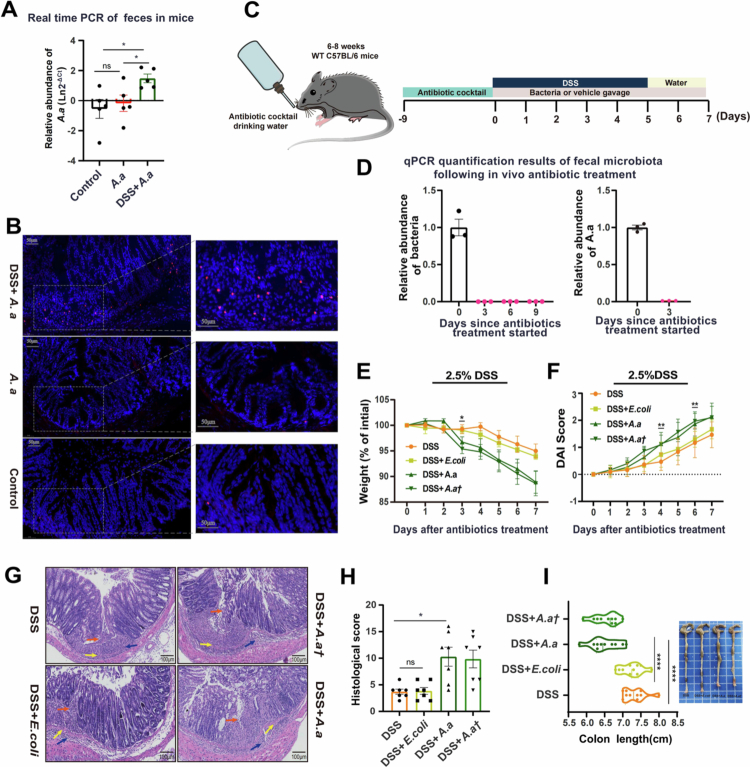
The relative abundance of *A. actinomycetemcomitans* was transiently detectable​ in the colon of antibiotic-treated mice and was associated with exacerbated colitis. (A) *A. actinomycetemcomitans* abundance in mice feces among the PBS control group, *A. actinomycetemcomitans* group, and DSS + *A. actinomycetemcomitans* group. (B) Representative FISH images of *A. actinomycetemcomitans* in the colons of the normal control group, DSS group and DSS + *A. actinomycetemcomitans* group using Cy3-conjugated probe (red: *A. actinomycetemcomitans* DNA). Scale bar: 50 μm. (C) Experimental design and timeline for the antibiotic (ABX) depletion model (*n* = 10 mice per group). A broad-spectrum antibiotic cocktail (0.2 g/L ampicillin, 0.2 g/L metronidazole, 0.2 g/L neomycin, and 0.1 g/L vancomycin) was added to the drinking water for 9 days. (D) qPCR analysis of total bacterial 16S rRNA gene copies in feces after antibiotic administration, confirming microbiota depletion. (E) Body weight and (F) disease activity index (DAI) of each group after antibiotics and DSS treatment. (G) Representative H&E-stained colon sections (scale bar: 100 μm). Arrow annotations are the same as those in Figure 2E. (H) Histological score indicating colitis severity. Scores were derived from the assessment of at least 3 longitudinally-oriented crypt-villus fields per mouse. (I) Colon length measured at the experimental endpoint. Data are presented as mean ± SEM and are representative of two independent experiments. Each symbol represents one mouse (*n* = 10 mice per group for panels where applicable). Compared to the level of variability with the DSS group using two-sided one-way ANOVA followed by the least significant difference (LSD) post–hoc test or two-sided unpaired Student’s t-test and Dunnett T3 (uneven variance). The Shapiro‒Wilk test was used for data normality, and the Levin test was used for homogeneity of variance. *p < 0.05, **p < 0.01, ***p < 0.001, ****p < 0.0001. A. *a†*: *A. actinomycetemcomitans* with streptomycin resistance (50 μg/ml).

To determine whether orally administered *A. actinomycetemcomitans* can be detected in the colon, we first quantified its abundance in the feces of mice that received the bacterium, with or without DSS co-treatment*.* The results showed that *A. actinomycetemcomitans* DNA was transiently detectable in the colon only when inflammation occurred (Figure 3A). This discovery was also supported by the FISH results (Figure 3B).

Although we detected increased transient intestinal persistence of *A. actinomycetemcomitans* might accelerate the development of colitis in mice, and this process is always accompanied by the presence of commensal microbes. To assess whether the presence of *A. actinomycetemcomitans* alone was sufficient to influence colitis, and antibiotic-pretreated mice were employed to investigate the involvement of only *A. actinomycetemcomitans* in the initiation and development of colitis (Figure 3C). The consumption of microbiota was confirmed by quantitative PCR (Figure 3D). The aseptic mice were then given daily oral administration of bacteria or the PBS vector while colitis was induced (Figure 3C). Similar to previous studies, the oral administration of *A. actinomycetemcomitans* exacerbated DSS-induced colitis in antibiotic-pretreated mice (Figure 3E–I).

### The reproductive expansion ability of *A. actinomycetemcomitans* in the inflamed colon was restricted

**Figure 4.**
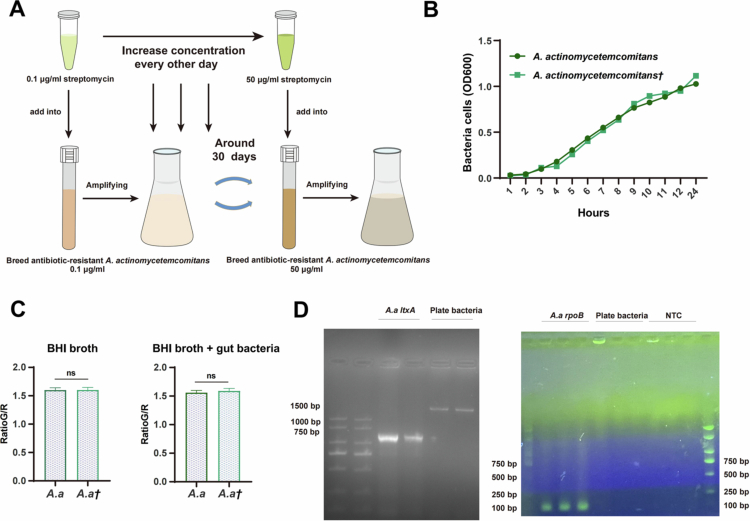
The capacity of *A. actinomycetemcomitans* to proliferate in an inflamed colon was restricted. (A) Schematic of the streptomycin resistance adaptation protocol for *A. actinomycetemcomitans*. (B) Growth curve of streptomycin-resistant *A. actinomycetemcomitans* and WT *A. actinomycetemcomitans*. Data points represent the mean optical density (OD600) ± SEM from three independent cultures. (C) Comparative analysis of total biomass (absorbance ratios) between streptomycin-adapted and non-adapted *A. actinomycetemcomitans*. Data are presented as the mean fluorescence ratio (green/red) ± SEM from three independent experiments. (D) Gel electrophoresis demonstrating the abundance of *A. actinomycetemcomitans* (*rpoB* and *ltxA* genes) in bacterial genomes extracted from fecal continuously diluted antibiotic plates. NTC: no template control.

To assess the survivability of oral bacteria, *A. actinomycetemcomitans* in the intestinal system, we domesticated *A. actinomycetemcomitans* with streptomycin resistance (Figure 4A). The growth curve and animal experiments revealed that antibiotic adaptation did not alter its growth characteristics or virulence (Figures 3 and 4). The total biomass remained unaltered post-amplification, irrespective of the presence or absence of the intestinal microbiota in the colitis model (Figure 4C).

Two days before the mice were sacrificed, the feces of the colitis mice gavaged with streptomycin-resistant *A. actinomycetemcomitans* were collected daily. The diluted homogenates were plated on streptomycin-containing BHI agar. After incubation for 48–96 h, no viable *A. actinomycetemcomitans* colonies were isolated, although other antibiotic-resistant bacteria were recovered (Figure S1A, S1B and Table S4). Total DNA was extracted from all the colonies harvested from the plate and amplified by PCR. Electrophoretic analysis of the amplicons failed to detect any *A. actinomycetemcomitans*-specific signal, confirming its absence from the cultured plate (Figure 4D). Together, the inability to isolate viable *A. actinomycetemcomitans* from feces despited detecting its DNA suggested that the bacterium did not expand substantially in the gut. The detectable DNA likely originated from non-viable bacterial cells or fragments that were transiting through the intestinal tract.

### Oral administration of *A. actinomycetemcomitans* was associated with intestinal barrier impairment and heightened inflammation in mice

**Figure 5.**
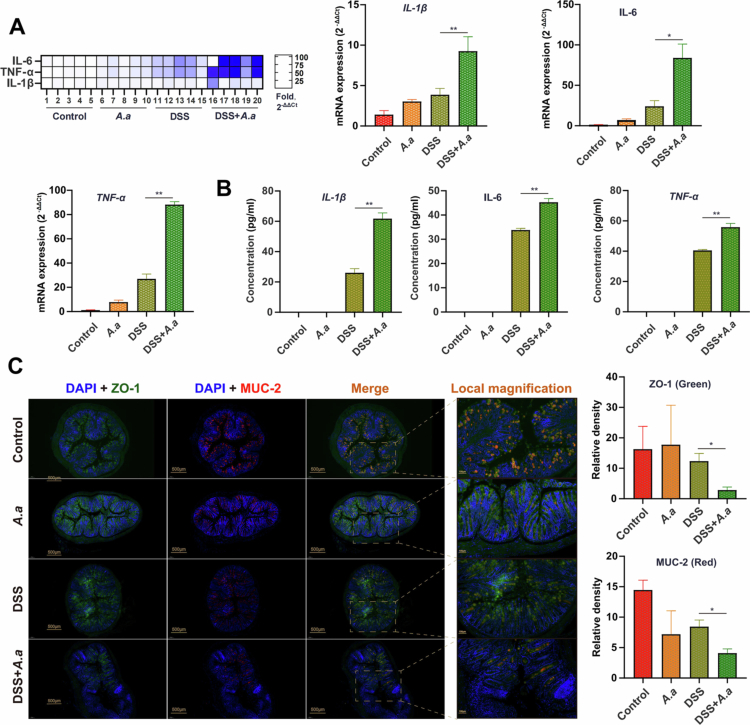
Oral administration of *A. actinomycetemcomitans* was associated with aggravated intestinal barrier impairment and elevated levels of inflammatory mediators in mice with colitis. (A) Relative mRNA expression levels of pro-inflammatory cytokines in colon tissue were normalized to *Gapdh* (2^-ΔΔCt^ method). (B) Serum concentrations of IL-6, IL-1β, and TNF-*α* measured by ELISA. (C) Representative immunofluorescence images of colon sections stained for the tight junction protein ZO-1 (green) and the mucin MUC-2 (red). Nuclei were counterstained with DAPI (blue). Scale bars: 200 μm. Quantification of ZO-1 and MUC-2 signal intensity was performed on at least 9 non-overlapping fields per mouse using ImageJ software. Data are representative of two independent experiments. Data are shown as mean ± SEM. Statistical analysis was performed using two-sided one-way ANOVA followed by the least significant difference (LSD) post–hoc test or two-sided unpaired Student’s t-test and Dunnett T3 (uneven variance). *p < 0.05, **p < 0.01, ***p < 0.001.

Dysfunctional immunity and chronic inflammation play a vital role in the development of IBD regulated by the intestinal microflora [11,36]. In order to investigate the immunoinflammatory response mediated by *A. actinomycetemcomitans*, we examined inflammatory markers in the colon tissue and blood of SPF mice treated with *A. actinomycetemcomitans*. The results showed that the concentrations of inflammatory factors in the colon tissue of colitis mice after oral gavage of *A. actinomycetemcomitans* were significantly higher than those in the DSS group (*Il-6*, *P* = 0.031; *Il-1β*, *P* = 0.005; *Tnf-α*, *P* < 0.0001) (Figure 5A). Similarly, the concentrations of inflammatory factors in the blood of colitis mice in the *A. actinomycetemcomitans* gavage group were also significantly higher than those in the DSS group (IL-6, *P* = 0.002; IL-1β, *P* = 0.002; TNF-*α*, *P* = 0.004) (Figure 5B).

Mechanical epithelial barrier dysfunction is a key pathogenic feature of IBD [37]. The barrier comprises a mucous layer and an epithelial cell layer [36]. Goblet cells secrete mucin-2 (MUC-2), the predominant mucosal protein, while tight junction proteins such as ZO-1 are crucial for barrier integrity [38]. In our study, treatment with *A. actinomycetemcomitans* was associated with reduced expression of both ZO-1 and MUC-2 in the colon (Figure 5C) compared to the control group. This suggested a compromise in barrier components. Consequently, the impairment of these epithelial and mucosal barrier elements may contribute to the exacerbated colitis observed following *A. actinomycetemcomitans* exposure.

### Oral gavage of *A. actinomycetemcomitans* altered the gut microbiota composition in mice

The intestinal microbiome serves as a critical interface between the external environment and the intestinal mucosa. Reduced microbial diversity and the resulting dysbiosis promote aberrant mucosal immune responses and tissue damage, thereby perpetuating disease [39]. Given this, we observed that the oral administration of *A. actinomycetemcomitans* in the absence of colitis led to reduced body weight and elevated DAI scores (Figure 2C and D), findings that are consistent with a potential alteration of the gut microbiota.

**Figure 6.**
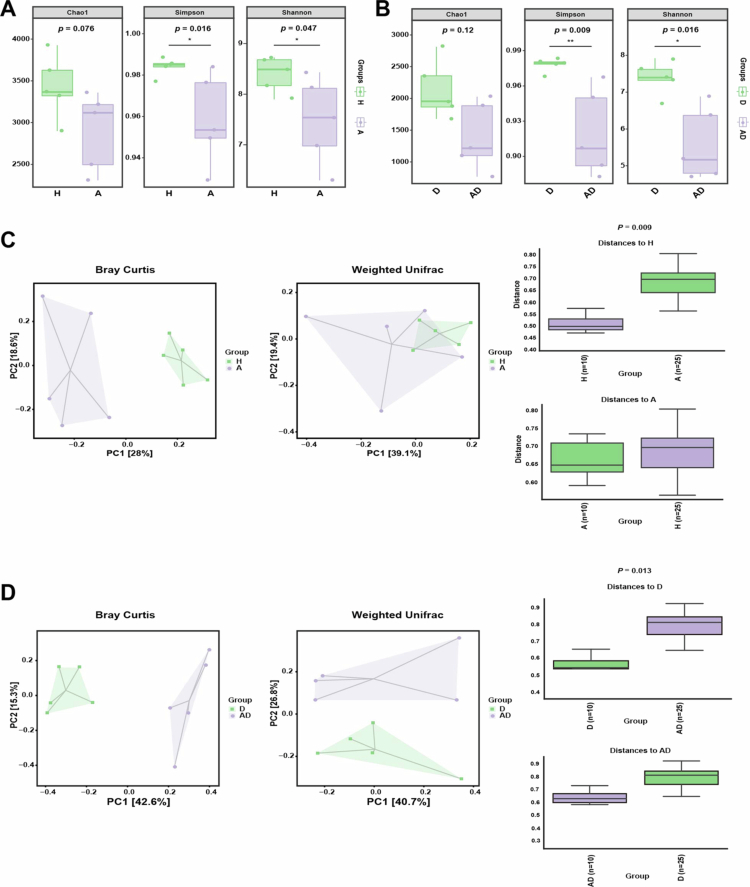
The gastrointestinal exposure of *A. actinomycetemcomitans* disrupted the intestinal flora in mice. The alpha diversity index (Chao1, Shannon and Simpson indices) between (A) groups H and A and between (B) groups D and AD are displayed, respectively. The number under the diversity index label is the *P*-value of two-sided Kruskal‒Wallis test. (C) and (D) showing the beta diversity indicators compared between groups H and A and between groups D and AD, respectively. Various algorithms have been adopted to reduce the multi-dimensional species data to one-dimensional data, namely, sample difference distances (Bray-Curtis and weighted UniFrac), so as to characterize the community differences between two samples from different perspectives. The ANOSIM algorithm tests the significance of differences among groups, and the significance of comparison is expressed as *p*-value. H: Health group; A: *A. actinomycetemcomitans* group; D: DSS group; AD: *A. actinomycetemcomitans* + DSS group.

We examined alterations in the intestinal microbiota of mice to assess the impact of gastrointestinal exposure of *A. actinomycetemcomitans* on microbial composition. The results demonstrated that *A. actinomycetemcomitans* gastrointestinal exposure reduced the alpha diversity of the gut microbiome in mice, regardless of DSS treatment. Specifically, the experimental group showed decreased community richness (Chao1 index; *p* = 0.012 and 0.016) and significantly lower species diversity (Shannon and Simpson indices; *p* < 0.05; Figure 6A and B). Beta diversity indices (Bray Curtis, Jaccard, weighted UniFrac, and unweighted UniFrac) were performed to evaluate the variations in intestinal microbial community structure and membership between groups (with or without *A. actinomycetemcomitans* gavage). Individuals in the control group tended to group together and to be significantly separated from those in the experimental group in PCoA plots based on these indicators (Figure 6C, D, and S2), indicating that intestinal exposure of *A. actinomycetemcomitans* did significantly alter the intestinal microbial composition in mice and was associated with alterations in the microbial community.

### Oral administration of *A. actinomycetemcomitans* in colitis mice was associated with an expansion of specific intestinal taxa and exacerbated disease

**Figure 7.**
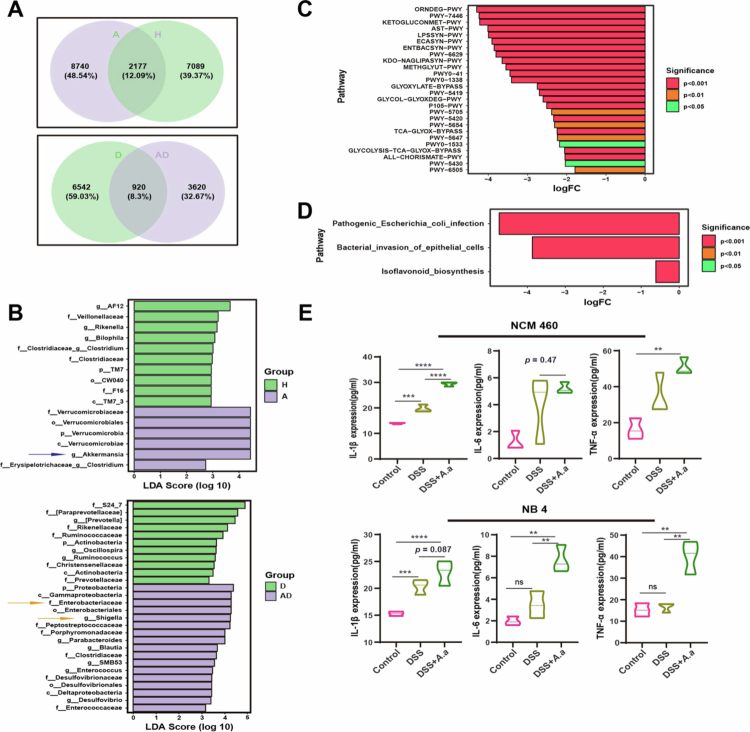
Oral administration of *A. actinomycetemcomitans* during colitis was associated with an expansion of potentially detrimental bacterial taxa. (A) The Venn diagrams for comparison between groups H and A and between groups D and AD are listed, respectively. (B) The histogram of LDA effect values of marker species between groups based on classification level tree are listed, respectively compared between groups H and A and between groups D and AD. (C) The difference in MetaCyc metabolic pathways between groups D and AD. No pathway was significantly different between groups H and A. The method of metagenomeSeq was used to obtain the abundance data of metabolic pathway to determine the significant differences in metabolic pathway between groups. The positive value of the horizontal axis logFC [log2 (fold change)] means that group D was up-regulated compared with group AD, and the negative value was down-regulated; the vertical coordinates are different MetaCyc metabolic pathway tags; different colors show the degree of significance. (D) Different metabolic pathways of KEGG between groups. The positive value of the horizontal axis logFC represents the up-regulation [log2 (fold change)] of group D compared with group AD, and the negative value is down-regulated; the vertical coordinates are different KEGG metabolic pathway tags; different colors show the degree of significance. (E) Detection of inflammatory factors in the culture supernatant of NB4 and NCM460 cells stimulated by intestinal bacterial extracts (1 μg/ml) for 24 hours. Data are from one experiment that is representative of two independent experiments. *p < 0.05, **p < 0.01, ***p < 0.001, by two-sided one-way ANOVA followed by LSD post hoc test or two-sided unpaired Student’s t-test and Dunnett T3 (uneven variance). The Shapiro‒Wilk test for data normality and the Levin test for homogeneity of variance were used. Differences in the mean ( ± SEM) are displayed. H: Health group; A: *A. actinomycetemcomitans* group; D: DSS group; AD: *A. actinomycetemcomitans* + DSS group.

Our data revealed significant alterations in the intestinal microbiome between the oral *A. actinomycetemcomitans* gavage group (experimental group) and the control group, although this analysis did not identify the specific taxa driving these changes. To further characterize the microbial shifts, we first quantified the proportion of shared and unique ASVs/OTUs between groups using a Venn diagram (Figure 7A). In the absence of DSS, only 12.09% of the ASVs/OTUs were shared between the experimental and control groups, whereas during colitis, this proportion dropped to 8.3%. Conversely, the experimental group harbored 48.54% and 32.67% unique ASVs/OTUs under these respective conditions, demonstrating an increase in the relative abundance of distinct taxa.

Additional LEfSe analysis was performed to identify particular bacteria linked to *A. actinomycetemcomitans* intestinal infection. In the presence of colitis, *Enterobacteriaceae* and *Shigella* were significantly enriched in the experimental group, whereas in the absence of colitis, although intragastric administration of *A. actinomycetemcomitans* resulted in the enrichment of *Verrucomicrobiaceae*, and the abundance of *Akkermansia* increased (Figure 7B and S3). *Akkermansia muciniphila* is generally considered a beneficial gut commensal bacterium associated with mucosal health. Its enrichment is associated with antagonistic pathogen protection, improved tight junction and intestinal permeability, reduced inflammation, and an enhanced adaptive immune response [40], as well as a limited scale of colitis [41]. Importantly, our results showed that *A. actinomycetemcomitans* did not induce intestinal injury in the absence of colitis (Figure 2E-2H).

Intestinal microorganisms can influence colonic inflammation through metabolic pathways [42,43]. Our metabolic functional pathway prediction showed that oral *A. actinomycetemcomitans* administration altered subsequent metabolic pathways in mice with colitis, but not in those without colitis (Figure 7C). KEGG pathway analysis revealed that the most significantly altered pathways were associated with *E. coli* infection and bacterial invasion of intestinal epithelial cells (Figure 7D). An examination of species contributions to MetaCyc metabolic pathways indicated that *Shigella* and *Enterobacteriaceae* – identified as differentially abundant taxa in the LEfSe analysis – contributed most substantially to these functional changes (Figure S4). To further investigate the functional impact of the altered microbiota, we isolated total bacteria from the fecal samples of *A. actinomycetemcomitans*-treated mice for subsequent *in vitro* assays [22,44]. In preliminary *in vitro* assays, the exposure of intestinal epithelial cells to sterile fecal extracts from *A. actinomycetemcomitans*-gavaged mice significantly increased the release of inflammatory cytokines (Figure 7E). These findings suggested that the exposure to *A. actinomycetemcomitans* was associated with an expansion of specific bacterial taxa and may contribute to exacerbated colitis, particularly in the context of pre-existing intestinal damage.

## Discussion

The discovery that poor oral hygiene disrupts the oral‒intestinal microbiota balance, contributing to IBD pathogenesis, dates back approximately a decade [45]. Subsequent studies identified specific oral or periodontal microbial species involved in this process [46,47]. Recent recognition of oral-systemic communication axes, particularly the oral-gut axis, has sparked widespread interest in oral dysbiosis as a disease modifier [10,48]. Large-scale fecal shotgun metagenomic sequencing has further substantiated the link between intestinal enrichment of oral/periodontal-associated bacteria and IBD [49]. Moreover, recent work has demonstrated that periodontitis-associated salivary bacteria can induce intestinal inflammation in both mice and humans, concomitant with gut microbiota dysbiosis [50]. However, the specific pathogenic bacterial strains that mediate these effects remain largely unexplored. Our study identified an association between the periodontal pathogen *A. actinomycetemcomitans* and IBD. To explore potential causal relationships, we employed a DSS-induced colitis mouse model. The experimental data indicated that exposure to *A. actinomycetemcomitans* exacerbated colonic inflammation by perturbing the microbiota, particularly under high-dose gavage conditions in specific DSS-induced colitis models.

The intestine harbors a small but significant fraction (~2%) of oral bacteria [9], among which *P. gingivalis*, *Fusobacterium nucleatum* (*F. nucleatum*), *Klebsiella spp.*, and *Campylobacter spp.* emerged as potential IBD contributors because of their immuno-inflammatory effects [15]. Recent advances have highlighted the intestinal expansion of periodontitis-associated bacteria as an IBD feature. *F. nucleatum*, a symbiotic periodontitis-associated pathogen in the intestinal and oral cavity, enriched and aggravated the inflammation in colitis mice as well as in patients with IBD [46,51,52], which may be related to the promotion of flora imbalance and metabolic disorders [53]. Meanwhile, *F. nucleatum* may worsen intestinal inflammation by controlling epithelial or immunological cells via virulence proteins or generating extracellular vesicles [51,54,55]. In addition, *P. gingivalis*, a pathogen associated with periodontitis, can reach the gastrointestinal tract and alter the microbiome [56,57], weakening intestinal barrier function and increasing the immune inflammatory response in a colitis mouse model [33], which is related to the peptidylarginine deiminase of bacteria [34]. While numerous studies have demonstrated an epidemiological link between oral *F. nucleatum* and colorectal diseases, direct clinical evidence tracking the pathogenic process of oral-derived *F. nucleatum* in human colorectal diseases remains limited [58,59]. In parallel, although *P. gingivalis* is a common oral resident, and its transient persistence within the damaged intestinal tract has not been documented.

As a member of HACEK (*Haemophilus*, *Aggregatibacter*, *Cardiobacterium*, *Eikenella* and *Kingella*) group, *A. actinomycetemcomitans* naturally colonizes the oral cavity but can also be isolated from various non-oral infections, including infective endocarditis [29,60]. This bacterium harbors a 14-gene tad (tight adherence) operon, which confers considerable colonization potential in diverse niches [20]. Although these features suggest that *A. actinomycetemcomitans* can colonize extra-oral sites, our study and the existing literature indicate that it does not achieve high abundance or stable colonization in the inflamed intestine. The transient presence observed in our model likely reflected the high bacterial load introduced by experimental gavage rather than the intrinsic colonization capacity. Importantly, we did not recover viable *A. actinomycetemcomitans* from feces, suggesting this presence may represent non-replicating bacterial remnants or DNA fragments rather than stable colonization. Thus, the observed aggravation of colitis was more likely attributable to acute perturbation of the host‒microbe interface during the window of bacterial exposure, potentially via indirect effects on the resident microbiota or host immune tone. More research is needed to evaluate the survival of *A. actinomycetemcomitans* in the intestinal system, as isolated live organisms from the oral cavity or the intestinal tract are not isolated events [46,47,49]. Specifically, for example, future studies should focus on meta-transcription analysis at the RNA level, which could describe metagenomic and gene expression levels, respectively, to evaluate the active and functional pathways of the pathogenicity of oral periodontal pathogens in the intestinal tract during IBD.

Despite being components of the digestive system, the oral cavity and colon harbor distinct bacterial communities and occupy separate ecological niches [49]. The enrichment of oral-associated bacteria in IBD-inflamed intestines suggests that some oral microbes can adapt to the gut environment, albeit under stringent conditions [61]. The acidic gastric barrier represents a major obstacle preventing oral microbes from reaching the lower intestine [62–64]. In addition, while inflammation significantly reduces intestinal colonization resistance, numerous different factors still make it difficult for oral bacteria to expand in the intestinal tract. For example, an insufficient abundance of toxic oral bacteria [17], native intestinal bacteria through niche and nutritional competition, or bacteria limited by metabolites or quorum sensing molecules, and host participation in colonization resistance, etc. [63–65]. Notably, non-bacterial species in the intestinal tract, such as fungi and viruses, can also have an impact on the bacterial ecological niche [39,66,67]. In fact, characterizing these restrictions and the impact of external variables, including drugs, is actually essential to comprehend the mechanism of action of periodontal pathogens in IBD.

Periodontal pathogens cause intestinal inflammation either directly through the oral‒intestinal axis or indirectly through the immune inflammatory response caused by their own or highly toxic products in the blood and the bone marrow deviation of hematopoietic progenitor cell differentiation, which aggravates the inflammation [68,69]. In our model, the oral administration of *A. actinomycetemcomitans* was followed by increased expression of pro-inflammatory cytokines (TNF-*α*, IL-6 and IL-1β), concurrent with reduced expression of barrier proteins (ZO-1 and MUC-2) and alterations in the composition of the intestinal flora in mice with colitis. These observations suggested that *A. actinomycetemcomitans* exposure may initiate or amplify a cascade of events leading to barrier dysfunction and dysbiosis in a susceptible host. Damage to the intestinal epithelium and mucus barrier results in the infiltration of harmful substances, including toxins, pathogens and antigens, from the lumen into the mucous membrane [36,37]. Consequently, the intact intestinal barrier limits the access of *A. actinomycetemcomitans* to the colonic tissue in the absence of pre-existing colitis (Figure 3A and B). The colon only had a local immune inflammatory reaction and microbiota disorder (Figures 5 and 6) but did not cause inflammation to escape and spread. When colitis occured, nevertheless, the intestinal barrier function was destroyed, and *A. actinomycetemcomitans* or its components may gain access to the incomplete barrier and play a poisonous role in the colon, exacerbating the degradation of the epithelial and mucus barriers (Figure 5C). With the escape of inflammatory factors, the concomitant disruption of the microbial barrier increased the proliferation of harmful bacteria and further increased the production of inflammatory markers (Figure 7E), potentially contributing to exacerbated colitis. However, this study did not include direct barrier function assays. Future research will prioritize the use of techniques such as the FITC-Dextran permeability assay or TEER measurements to provide more direct evidence.

Periodontal pathogens accumulate in the oral cavity and migrate to the inflammatory intestinal tract, where colonization resistance is compromised. Subsequent alterations, such as microbial proliferation and community structure disruption, virulence factors, toxic metabolites, and other pro-inflammatory variables, thus aggravate colitis [15,36,49]. However, a limitation of this study is that it does not allow us to definitively rule out the contribution of bacterially secreted soluble toxins or cell wall components during the early stages or as cooperative contributors. The precise link between increased inflammatory factor expression in the colon and microbial barrier dysfunction is unknown. However, our findings showed that the inflammatory environment of the colon does not allow *A. actinomycetemcomitans* to benefit from selective proliferation, but rather increases the inflammatory phenotype and barrier breakdown. We speculate that *A. actinomycetemcomitans* had limited potential to compete for ecological niches with species in the gut, as well as its own activities. Hence, our additional research will need to clarify whether *A. actinomycetemcomitans* act directly on intestinal epithelial cells via virulence proteins to modify barrier function while causing harmful gut bacteria to proliferate.

In summary, our findings indicated that the oral administration of *A. actinomycetemcomitans* exacerbated colonic inflammation and perturbed the gut microbiota in a DSS-induced murine model. The interplay between the dysbiotic microbiota and dysregulated immunity represents a central pathogenic mechanism. Managing periodontitis and its associated microbial dysbiosis may thus represent a novel therapeutic approach for mitigating the progression of IBD. Future studies are warranted to translate these findings into clinical benefits for patients.

This study has several other limitations that must be acknowledged. First and foremost, the human cohort analysis is exploratory. The lack of detailed IBD subtyping, periodontal health data, comprehensive medication histories, oral health metrics, and matched controls limits any causal interpretation of the observed association between *A. actinomycetemcomitans* and IBD. These unmeasured confounders preclude definitive conclusions regarding the directionality or mechanism of this association. The findings should therefore be interpreted strictly as generating a hypothesis—that this specific oral pathobiont may be relevant in the gut environment of some IBD patients—which requires validation in larger, prospectively designed cohorts with rich phenotypic data. Second, methodological constraints warrant consideration. Our microbial taxonomic analysis relied on an older version of the Greengenes database. However, once standard, its outdated framework and limited coverage of novel taxa may have introduced annotation biases, particularly for the differential taxa highlighted in our analysis, potentially affecting the reliability of our screening results. More contemporary and curated databases (e.g. SILVA) would offer improved accuracy for future work. Third, we acknowledge limitations in contamination control. This study lacked systematic negative controls and standardized contamination screening protocols, which are indispensable for validating results from gnotobiotic-adjacent experiments. Although sterile techniques were employed, the absence of these data limits our ability to fully rule out exogenous contamination. Finally, the relatively small sample size in our experimental model may have limited statistical power, reducing the sensitivity to detect true differences after rigorous multiple comparison correction. To address these points, future studies will employ updated, cross-validated taxonomic databases, incorporate rigorous negative control strategies, and expand sample sizes to enhance the robustness, reliability, and representativeness of the conclusions.

## Conclusions

• IBD has become a global problem, and its pathogenesis is not completely clear. Periodontitis is common in the general population and may be linked to IBD via periodontal pathogens.
• Our study demonstrated that oral administration of the periodontal pathobiont *A. actinomycetemcomitans* exacerbated disease severity in a DSS-induced murine colitis model. This exacerbation was associated with transient detection of bacterial DNA in the colon, heightened inflammatory responses, impaired intestinal barrier integrity, and significant alterations in the gut microbiota composition. Crucially, we did not recover viable *A. actinomycetemcomitans* from feces, and it did not establish stable colonization. Therefore, the observed effects were more likely attributable to acute perturbation of the gut ecosystem following high-dose exposure, rather than direct, sustained pathogenicity. These findings contribute to the understanding of oral‒gut axis interactions and suggest that periodontal microbes may influence distal intestinal inflammation through community-level effects. Further research is needed to define the relevance of this mechanism in human IBD.
• We can actively target certain immune microbial indicators to lessen the impact on IBD by identifying and isolating definite periodontal pathogens that damage the intestinal tract and researching their pathogenic process.

## Supplementary Material

Supplementary_materials.docx Supplementary_materials.docx

β diversity.xls β diversity.xls

Basic Information of non-IBD Control and IBD Patients.xlsx Basic Information of non-IBD Control and IBD Patients.xlsx

Statistics.xlsx Statistics.xlsx

List of LEfSe Statistical Results.xls List of LEfSe Statistical Results.xls

a diversity.xls a diversity.xls

## Data Availability

The raw data supporting the conclusions of this article will be made available by the authors without undue reservation to any qualified researcher. The raw 16S rRNA gene sequencing data generated in this study have been deposited in the NCBI Sequence Read Archive (SRA) under the BioProject accession number PRJNA1353878.
