# Supplementary material for: Aggregatibacter actinomycetemcomitans exacerbates colitis and perturbs the gut microbiota in a murine model​
Source: J Oral Microbiol. 2026 Jan 11;18(1):2613536. doi: 10.1080/20002297.2026.2613536 (PMC12794699; doi:10.1080/20002297.2026.2613536)
Supplement: Supplementary_materials.docx [file ZJOM_A_2613536_SM5170.docx]

**Supplementary materials**

*Aggregatibacter actinomycetemcomitans* exacerbates colitis and perturbs the gut microbiota in a murine model​

Xu Chen^1,2,3*^, Xiaoming Zhu^1,2,3,5*,^ Yiran Liu^1,3,4^, Lu Ma^1,2,3^, Lu Li^1,2,3^, Jitong Dong^1^,

Zirui Li^1,3^, Tianyao Wang^1,3^, Juan Zhang^1,3^, Yan Xu^1,2,3※^

^
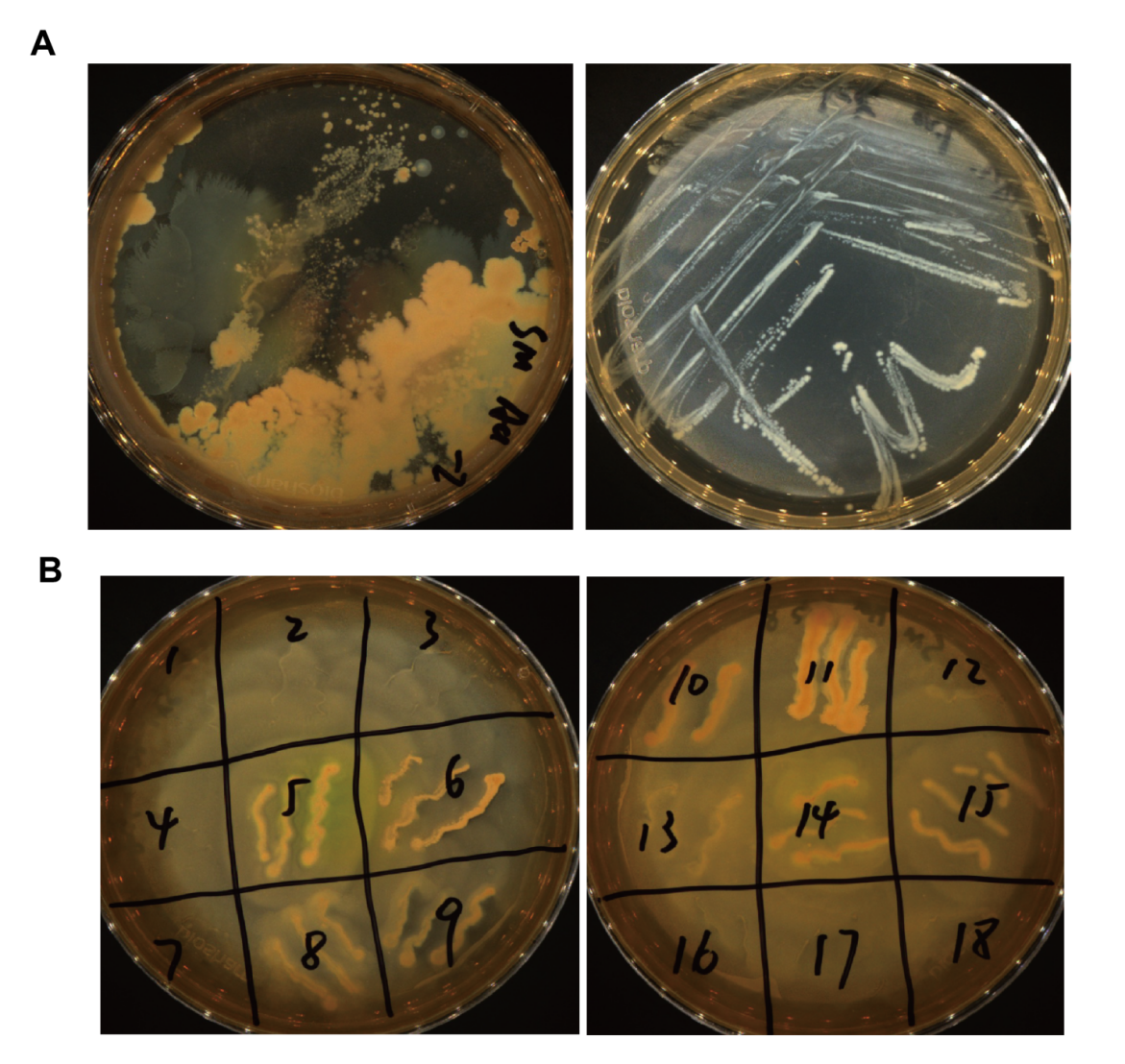
^

**Fig. S1** (A) Left: Landmark plate coating map following gradient dilution of feces in streptomycin resistant *A. actinomycetemcomitans* gavage group. Right: streptomycin resistant *A. actinomycetemcomitans* growth plate. (B) Amplification of monoclonal colonies selected from continuously diluted antibiotic plates of feces cultured for 48-72 hours.

**
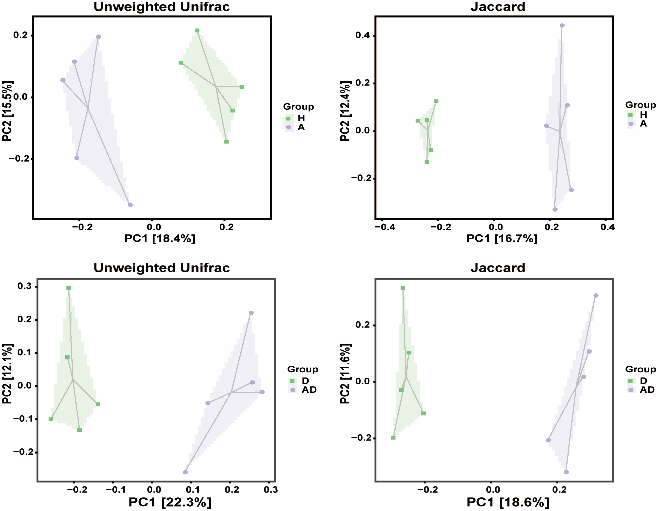
**

**Fig. S2** The beta diversity indicators compared between group H and A, group D and AD respectively. Various algorithms have been adopted to reduce the multi-dimensional species data to one dimensional data, namely, sample difference distance (Jaccard and Unweighted UniFrac), so as to characterize the community differences between the two samples from different perspectives.


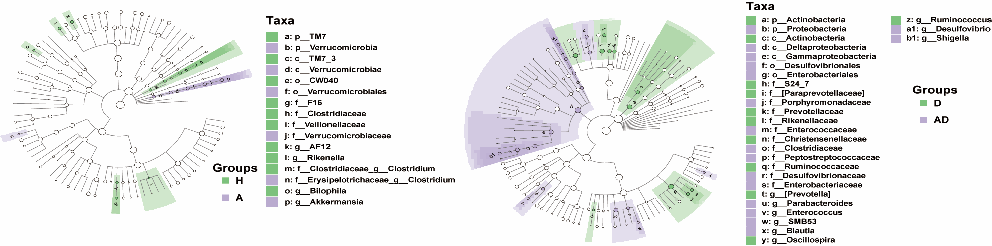


**Fig. S3** The histogram of LDA effect values of marker species and the display diagram of taxon differences between groups based on classification level tree are listed respectively compared between group H and A, group D and AD.


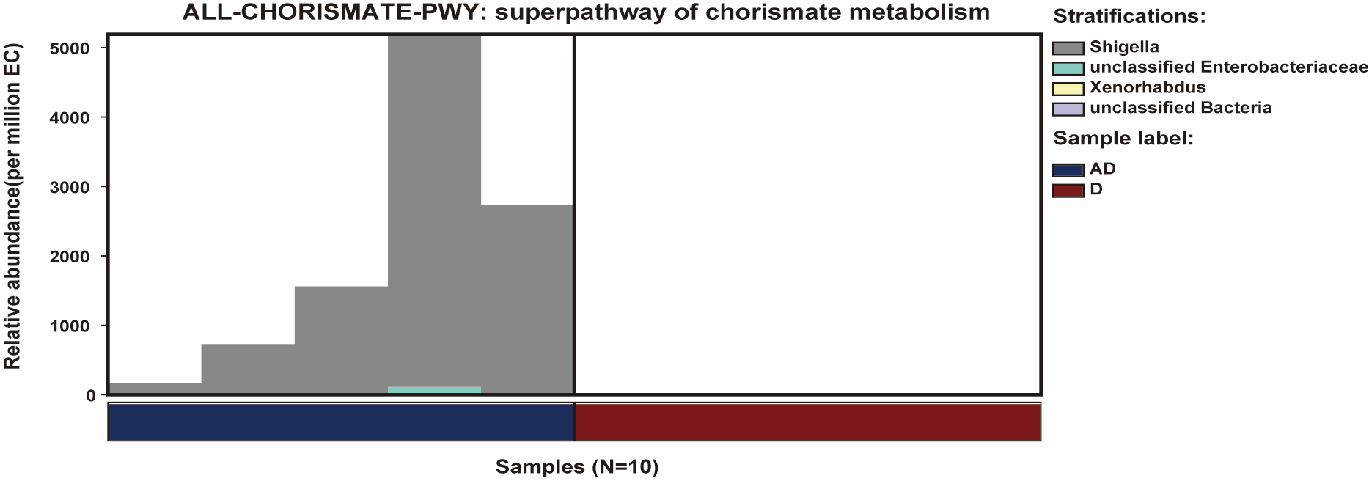


**Fig. S4** Species composition map of differential MetaCyc metabolic pathway. Abscissa is different sample label, ordinate is the relative abundance of metabolic pathway, and different colors are used to show the contribution of different taxon species to the metabolic pathway at the same classification level (the default is genus level).


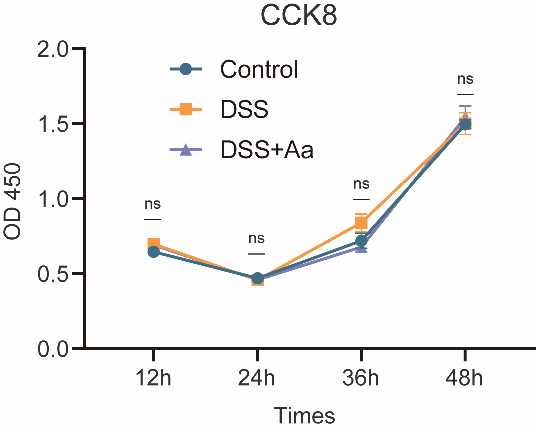


**Fig. S5** The proliferation curve over time following stimulation of cells with bacterial lysates (1 μg/ml). The data are presented as the mean difference (±SEM). Levene's

test was used to assess the homogeneity of variance. One-way ANOVA (with LSD post hoc test) was employed for comparisons, and ns indicates no significant difference.

**Table S1.**  **Basic Information of non-IBD Control and IBD Patients.**

| **Characteristics** | **non-IBD (n=21)** | | **IBD (n=28)** | | **P value** |
| --- | --- | --- | --- | --- | --- |
| **Age** |  |  |  |  |  |
| <=50 | 19 | 90.48% | 23 | 82.14% | 0.683 |
| >50 | 2 | 9.52% | 5 | 17.86% |  |
| **Gender** |  |  |  |  |  |
| M | 6 | 28.57% | 17 | 60.71% | 0.042 |
| F | 15 | 71.43% | 11 | 39.29% |  |

**Table S2. Primer sequences**

| Gene name | Primer | Sequence |
| --- | --- | --- |
| Mouse TNF-α | Forward | CTGAACTTCGGGGTGATCGG |
|  | Reverse | GGCTTGTCACTCGAATTTTGAGA |
| Mouse IL-1β | Forward | GAAATGCCACCTTTTGACAGTG |
|  | Reverse | TGGATGCTCTCATCAGGACAG |
| Mouse IL-6 | Forward | CTGCAAGAGACTTCCATCCAG |
|  | Reverse | AGTGGTATAGACAGGTCTGTTGG |
| Mouse Gapdh | Forward | CCCTTAAGAGGGATGCTGCC |
|  | Reverse | TACGGCCAAATCCGTTCACA |
| Universal QPCR  bacterial primer | Forward | ACTCCTACGGGAGGCAGCAGT |
|  | Reverse | ATTACCGCGGCTGCTGGC |
| *A. actinomycetemcomitans rpoB* | Forward | TCACCAAGAAGTGAATGCAGGCGAAACC |
|  | Reverse | ACGATGTCCACGAATTTCACGAAACCGC |
| *A. actinomycetemcomitans ltxA* | Forward | GCCGACACCAAAGACAAAGTCT |
|  | Reverse | GCCCATAACCAAGCCACATAC |
| 16s rRNA V1-V9 | Forward | AGAGTTTGATCCTGGCTCAG |
|  | Reverse | GGTTACCTTGTTACGACTT |

**Table S3 Disease activity index (DAI) scoring system**

| **Weight loss(% of intial )** | **Stool**  **consistency** | **Occult/gross rectal**  **bleeding** | **DAI score** |
| --- | --- | --- | --- |
| 0 | Normal | Negative hemoccult | 0 |
| 1-5 | Soft but still formed | Positive hemoccult | 1 |
| 5-10 | Semi-sparse stool | Bloody stool | 2 |
| 10-20 | Diarrhoea or sparse stool | Rectal bleeding | 3 |
| >20 | / | / | 4 |

Note: The normal stool is dry and granular, the semi-sparse stool is paste but does not stick to the anus, the sparse stool is liquid and sticky. Scores were tallied for each category and then divided by three to obtain the DAI.

**Table S4 Blast results of single bacterial colony**

| **Bacteria genus** | **Colony number** |
| --- | --- |
| Proteus spp. | 1、2、5、7、14-17 |
| Achromobacter spp. | 8-10 |
| Parabacteroides spp. | 6 |
| Acinetobacter spp. | 11 |
| Enterococcus spp. | 18 |
| Unidentified | 3、4、12、13 |

**Table S5 Statistics table of sequencing depth per sample**

| **SampleID** | **Input** | **Filtered** | **Denoised** | **Merged** | **Non-chimeric** | **Non-singleton** |
| --- | --- | --- | --- | --- | --- | --- |
| H1 | 102615 | 98757 | 96463 | 86104 | 49952 | 47301 |
| H2 | 97372 | 90976 | 89087 | 82905 | 47294 | 45752 |
| H3 | 105701 | 101138 | 99263 | 91228 | 46153 | 43064 |
| H4 | 89884 | 85919 | 83796 | 75224 | 43025 | 40943 |
| H5 | 114643 | 109753 | 107332 | 98322 | 55955 | 54068 |
| A1 | 108283 | 100610 | 99056 | 93972 | 56562 | 55409 |
| A2 | 99188 | 93834 | 91707 | 84284 | 54031 | 51974 |
| A3 | 93815 | 89376 | 87846 | 82679 | 55274 | 54218 |
| A4 | 92307 | 86302 | 84628 | 79550 | 48045 | 45818 |
| A5 | 92531 | 84971 | 83177 | 76892 | 46470 | 44669 |
| D1 | 95237 | 88544 | 86817 | 82127 | 50134 | 49180 |
| D2 | 102830 | 97333 | 95389 | 89038 | 48222 | 45903 |
| D3 | 103295 | 98582 | 96291 | 85321 | 51260 | 50130 |
| D4 | 96571 | 90774 | 89460 | 85444 | 47626 | 46528 |
| D5 | 91215 | 88009 | 85518 | 76325 | 45122 | 43634 |
| AD1 | 85718 | 82328 | 81155 | 77241 | 51123 | 50110 |
| AD2 | 104609 | 100627 | 98095 | 90525 | 54922 | 53773 |
| AD3 | 90261 | 85273 | 84270 | 80158 | 62362 | 62111 |
| AD4 | 100124 | 96235 | 94918 | 88121 | 58163 | 57636 |
| AD5 | 98059 | 94567 | 93437 | 89144 | 67688 | 67322 |

**Supplementary Methods**

**Bacterial growth curve determination**

For the determination of bacterial growth curves, absorb about 1 ml of bacterial culture broth into a conical flask containing 50 ml of broth and shake well. Its OD_600_ value was measured. The reading at this time is the zero-hour reading value in the strain growth curve. Shake bacterial broth (230 rpm) at constant temperature of 37 °C. Remove the conical bottle from the shaker every 30-60 minutes, absorb about 2 ml bacteria solution in the quartz colorimetric plate on the super clean table, and read the OD_600_ value with the photoelectric colorimeter. Record the data measured each time. The growth curve under the experimental circumstances is constructed by taking the observed time as the abscissa and the logarithm of the number of bacteria (OD_600_) as the ordinate.

The growth curves were measured separately, in this case, they are in the logarithmic phase of their growth. Correspondingly, the standard curve associated to plate diffusion CFU and optical density (OD) was constructed at 600nm, and the absorbance value of OD_600_ was used to estimate the number of CFUs of cultured bacteria. Under the above-mentioned culture and growth conditions, when OD_600_=0.1, the bacterial concentration of *A. actinomycetemcomitans* is 0.83×10^8^ CFU·mL^−1^, while *E. coli* is 0.1×10^9^ CFU·mL^−1^.

**16S rRNA amplicon sequencing and analysis.**

16S rRNA gene sequencing was conducted by PANOMIX Biomedical Tech Co., Ltd. (Suzhou, China). Fresh mouse fecal samples were collected for 16S rRNA gene amplicon sequencing. Following genomic DNA extraction, quality control was performed using a Thermo NanoDrop 2000 UV microspectrophotometer and 1% agarose gel electrophoresis. Specific primers were used for PCR amplification, and products were purified using the TruSeq Nano DNA LT Library Prep Kit (Illumina). After library construction and quality control, quantification and normalization were carried out using Qubit. The libraries were sequenced on the Illumina MiSeq/NovaSeq platform.

The QIIME2 pipeline (2023.9) was employed for processing and filtering multiplexed sequence reads. Operational Taxonomic Units (OTUs) were clustered at 97% similarity threshold. Taxonomic assignment of representative sequences was performed against the Greengenes database (Release 13.8). Chimeric sequences were identified and removed using the UCHIME algorithm implemented in USEARCH (v7.0.1090). An OTU abundance table was generated based on sequence counts for subsequent analysis.

OTU profiling, alpha/beta diversity analyses, and rank abundance curves were conducted using QIIME2 (2023.9). Differential species and functional features were identified using LEfSe and Wilcoxon rank-sum test (R version 4.0.1). The detailed information regarding quality filtering thresholds, read trimming/truncation parameters, and denoising settings applied in this study is provided in the table S5.
